# Type 1 diabetes associated with other autoimmune diseases: is there any association with glycemic control and microvascular complications?

**DOI:** 10.1186/1758-5996-7-S1-A88

**Published:** 2015-11-11

**Authors:** Ana Luíza Campanholo, Isabella Albuquerque Pinto Rebello, Karina Tabet Munoz, Joana Rodrigues Dantas Pereira, Marcus Miranda dos Santos Oliveira, Lenita Zajdenverg, Melanie Rodacki

**Affiliations:** 1 Hospital Universitario Clementino Fraga Filho, Rio de Janeiro, Brazil

## Background

Patients with type 1 diabetes (T1D) may develop additional autoimmune diseases. However, it is not clear whether this is associated with a worse metabolic control or more frequent microvascular complications.

## Objective

To evaluate if T1D patients with additional autoimmune diseases have differences in the glycemic control and microvascular complications compared with patients solely with T1D.

## Materials and methods

This is an observational cross-sectional study. Patients with T1D associated with another autoimmune disorder were selected from all the T1D patients that regularly attended the outpatient clinic of the Diabetes unit. A group of patients of the same outpatient clinic without those disorders was randomly selected as a control group. The presence of retinopathy, nephropathy and last hemoglobin A1C (HbA1C) were retrieved from medical charts. The data were analyzed at SPSS using U test Mann Whitney for continuous variables and chi square test for nominal variables. A p value of 0.05 was stablished.

## Results

36 patients out of 374 T1D patients were found to have other autoimmune disorders and 39 without those diseases were included as the control group. The mean age and T1D duration were 28.8 (±10) and 17.9 (±6) yrs., respectively. 63% were females and 37% males. The mean duration of diabetes was 17.56 yrs. in the autoimmune disorders group and 18.31 yrs. in the control group, without statistical difference (p=0.451). The frequency of other autoimmune diseases were as follows: hypothyroidism (61.1%), hyperthyroidism (16.7%), rheumatoid arthritis (2.8%), scleroderma (2.8%), celiac disease (5.6%), ulcerative colitis (2.8%), polyglandular syndrome (2.8%), autoimmune cirrhosis (2.8%), dermatomyositis (2.8%). Patients with other autoimmune disorders had higher HbA1C levels than controls (8.5% ±1.54 x 7.66% ±1.22, p=0.005), but did not show differences in the prevalence of nephropathy or retinopathy (p=0.14 and 0.15, respectively).

## Conclusion

This study indicates that patients with T1D and additional autoimmune diseases have a poorer glycemic control, with higher levels of HbA1C, but no differences in the frequency of diabetes complications. Further longitudinal studies should be performed to identify if the worse glycemic control will lead to a higher frequency of microvascular complications over time.

